# Predicting the clinical trajectory in critically ill patients with sepsis: a cohort study

**DOI:** 10.1186/s13054-019-2687-z

**Published:** 2019-12-12

**Authors:** Peter M. C. Klein Klouwenberg, Cristian Spitoni, Tom van der Poll, Marc J. Bonten, Olaf L. Cremer, on behalf of the MARS consortium, Jos F. Frencken, Kirsten van de Groep, Marlies E. Koster-Brouwer, David S. Y. Ong, Diana Verboom, Friso M. de Beer, Lieuwe D. J. Bos, Gerie J. Glas, Roosmarijn T. M. van Hooijdonk, Laura R. A. Schouten, Marleen Straat, Esther Witteveen, Luuk Wieske, Arie J. Hoogendijk, Mischa A. Huson, Lonneke A. van Vught

**Affiliations:** 1 Department of Medical Microbiology and Immunology, Rijnstate Hospital, Wagnerlaan 55, 6815 AD Arnhem, the Netherlands; 2 Department of Mathematics, University Utrecht, Utrecht, the Netherlands; 3 Center for Experimental and Molecular Medicine, Division of Infectious Diseases, Amsterdam University Medical Centers, location Academic Medical Center, University of Amsterdam, Amsterdam, the Netherlands; 4 Department of Medical Microbiology, University Medical Center Utrecht, Utrecht University, Utrecht, the Netherlands; 5 Julius Center for Health Sciences and Primary Care, University Medical Center Utrecht, Utrecht University, Utrecht, the Netherlands; 6 Department of Intensive Care Medicine, University Medical Center Utrecht, Utrecht University, Utrecht, the Netherlands

**Keywords:** Intensive care unit, Epidemiology, Outcome, Organ failure, Sepsis, Markov model

## Abstract

**Background:**

To develop a mathematical model to estimate daily evolution of disease severity using routinely available parameters in patients admitted to the intensive care unit (ICU).

**Methods:**

Over a 3-year period, we prospectively enrolled consecutive adults with sepsis and categorized patients as (1) being at risk for developing (more severe) organ dysfunction, (2) having (potentially still reversible) limited organ failure, or (3) having multiple-organ failure. Daily probabilities for transitions between these disease states, and to death or discharge, during the first 2 weeks in ICU were calculated using a multi-state model that was updated every 2 days using both baseline and time-varying information. The model was validated in independent patients.

**Results:**

We studied 1371 sepsis admissions in 1251 patients. Upon presentation, 53 (4%) were classed at risk, 1151 (84%) had limited organ failure, and 167 (12%) had multiple-organ failure. Among patients with limited organ failure, 197 (17%) evolved to multiple-organ failure or died and 809 (70%) improved or were discharged alive within 14 days. Among patients with multiple-organ failure, 67 (40%) died and 91 (54%) improved or were discharged. Treatment response could be predicted with reasonable accuracy (c-statistic ranging from 0.55 to 0.81 for individual disease states, and 0.67 overall). Model performance in the validation cohort was similar.

**Conclusions:**

This prediction model that estimates daily evolution of disease severity during sepsis may eventually support clinicians in making better informed treatment decisions and could be used to evaluate prognostic biomarkers or perform in silico modeling of novel sepsis therapies during trial design.

**Clinical trial registration:**

ClinicalTrials.gov NCT01905033

## Background

Sepsis is defined by life-threatening organ dysfunction due to a dysregulated host response to infection [1]. The current sepsis-3 definitions help early recognition of infected patients who are prone to develop a complicated course in emergency departments and general wards, but they do not predict the clinical response once initial resuscitation and organ support in the ICU have been provided. In fact, in patients with organ dysfunction or shock of recent onset, averting the progression of these—potentially still reversible—abnormalities is the main goal of critical care providers. Unfortunately, it is very difficult for clinicians to predict at the bedside which patients will respond favorably to their interventions, and who will deteriorate despite all resuscitative efforts. Current prognostic models for ICU patients such as the Acute Physiology and Chronic Health Evaluation (APACHE) score include only admission data and thus cannot be updated during the course of the disease.

We therefore developed and validated a model that uses daily information about the clinical condition of individual sepsis patients to make updated predictions regarding disease progression, by estimating the transitions between three intermediate states (i.e., different levels of organ failure) as well as towards two absorbing states (i.e., death and discharge) during the first 14 days in ICU.

## Methods

### Study design and population

This work was part of the Molecular Diagnosis and Risk Stratification of Sepsis (MARS) project, a prospective cohort study performed in the mixed ICUs of two tertiary referral centers in the Netherlands between January 2011 and December 2013 (ClinicalTrials.gov identifier NCT01905033) [2]. The Institutional Review Board approved an opt-out method of enrolment (IRB number 10-056C) whereby participants and family members were notified of the study by a brochure with an attached opt-out card that was provided at ICU admission. For model derivation, we analyzed all adults with sepsis as their main reason for presentation who had been admitted to ICU for ≥ 24 h. For patients in whom life support was ultimately withdrawn, we excluded all events following the moment that end-of-life care was initiated (i.e., ICU days until this time point were used for model fitting, but observation time was subsequently censored) for those patients who were discharged alive. Any readmissions occurring within 24 h of ICU discharge were merged and considered continuous with the previous admission period. For model validation, we analyzed an additional cohort of patients who presented to the UMC Utrecht between January 2014 and September 2016, using identical inclusion criteria.

### Classification of organ dysfunction

Since all patients fulfilled basic criteria for organ dysfunction according to sepsis-3 definitions, we sought to provide further prognostic stratification based on the number, extent, and potential reversibility of organ failures (Table 1). For this, we considered several clinical features and laboratory variables that are beyond the scope of “simple” SOFA criteria. For instance, all patients requiring vasopressor infusions and having elevated serum lactate levels > 2 mmol/L were considered to have cardiovascular dysfunction, yet only patients with more severe circulatory abnormalities were considered to have refractory shock. Likewise, we included a gastro-intestinal failure score as an extra indicator of disease severity. To reflect potential reversibility of organ dysfunction, we incorporated the duration of symptoms in our definitions. For instance, oliguria or hypotension lasting only a few hours would indicate a risk of organ failure, whereas oliguria or hypotension that lasted for > 1 day was regarded to be a marker of established organ failure. We used the terms “no dysfunction,” “moderate dysfunction,” and “severe dysfunction” to indicate failure at the organ level. We subsequently classed patients as (1) being at risk for organ failure, (2) having limited organ failure, or (3) having multiple-organ failure (Table 2). Since the “at risk” category was defined as “moderate dysfunctions of limited duration in ≤ 2 organ systems,” all patients who were admitted in the “at risk” category actually also fulfilled the sepsis-3 definition (e.g., when organ failure was limited to mechanical ventilation for short durations, patients fulfilled both “at risk” and sepsis-3 definitions).

**Table 1:** Classification of new-onset organ failure

	No dysfunction	Moderate dysfunction	Severe dysfunction
Central nervous system	Awake and non-delirious	Delirium (positive CAM-ICU score on ≥ 1 observation) *or* use of continuous sedation includes (but not limited to) the infusion of propofol and midazolam at any dose	Prolonged coma (unresponsiveness to verbal commands, both with or without the use of continuous intravenous sedation (RASS ≤ − 4 or GCS ≤ 8) for > 24 h)
Cardiovascular	Hemodynamic stability without support	Arterial hypotension (SBP < 90 mmHg for > 2 h) *or* use of inotropes and vasopressors (continuous infusion of dobutamine, milrinone, and (nor)epinephrine at any dose or a serum lactate level > 2 mmol/L) *or* positive fluid balance (cumulative fluid intake minus output > 2 L/24 h)	Shock (use of high-dose vasopressors including (nor)epinephrine at > 0.1 μg/kg/min or arginine vasopressin at any dose for > 12 h, with concurrent positive fluid balances > 2 L/24 h and lactatemia > 2 mmol/L)
Respiratory	Spontaneous breathing without hypoxemia	Mild arterial hypoxemia (use of mechanical ventilation with a P/F ratio < 300 and PEEP > 5 cm H_2_O)	Severe arterial hypoxemia (P/F ratio < 200 despite mechanical ventilation with PEEP > 8 cm H_2_O)
Renal	Adequate diuresis with preserved GFR	Acute oliguria (urine output < 0.5 ml/kg/h for > 6 h, or < 500 ml per day) *or* GFR decrease > 50% (> 1.5-fold increase in serum creatinine from baseline)	Prolonged oliguria/anuria (urine output < 0.3 ml/kg/h for > 24 h, or < 200 ml per day) *or* GFR decrease > 75% (> 3-fold increase in serum creatinine from baseline, a single creatinine level > 350 μmol/L with an acute rise of > 44 μmol/L, *or* use of renal replacement therapy)
Coagulation	Normal hemostasis	Mild thrombocytopenia (platelet count < 100,000/μL) *or* abnormal coagulation (INR > 1.5 or APTT > 60 s)	Severe thrombocytopenia (platelet count < 50,000/μL)
Liver	Normal liver function	Mild hyperbilirubinemia (plasma total bilirubin > 30 μmol/L) *or* abnormal protein synthesis (plasma albumin concentration < 20 g/L) *or* mild transaminitis (AST or ALT blood levels > 500 U/L)	Severe hyperbilirubinemia (plasma total bilirubin > 100 μmol/L) *or* severe transaminitis (AST or ALT blood levels > 1000 U/L) *or* deficient protein synthesis (plasma albumin concentration < 15 g/L)
Gastro-intestinal	Normal gut function	Impaired enteral feeding (daily caloric intake < 50% of calculated needs)	Prolonged food intolerance (inability to provide enteral feeding due to high gastric aspirate volume, vomiting, bowel distension, severe diarrhea, intraabdominal hypertension or abdominal compartment syndrome for > 24 h)

In cases where definitions were not mutually exclusive, the worst level of organ dysfunction was assigned

*Abbreviations*: *ALT* alanine transaminase, *APTT* activated partial thromboplastin time, *AST* aspartate transferase, *CAM-ICU* confusion assessment method for the intensive care unit, *GCS* Glasgow coma scale, *GFR* glomerular filtration rate, *INR* international normalized ratio, *PEEP* positive end-expiratory pressure, *P/F* partial pressure arterial oxygen and fraction of inspired oxygen, *RASS* Richmond agitation sedation scale, *SBP* systolic blood pressure

**Table 2:** Classification of organ failure on the patient level

At risk	Limited organ failure	Multiple-organ failure
No organ dysfunctions or moderate dysfunctions in ≤ 2 organ systems	Moderate dysfunctions in ≤ 3 organ systems or severe dysfunctions in ≤ 2 organ systems	Severe dysfunctions in ≥ 3 organ systems

### Prognostic variables

Potential predictor variables were a priori selected and classified according to the Prediction-Infection-Response-Organ dysfunction (PIRO) system [3, 4]. These encompassed both baseline (time-fixed) and daily (time-varying) variables, including (P) predisposing factors (i.e., age, gender, immunodeficiency, cardiovascular disease, respiratory insufficiency, renal insufficiency, diabetes mellitus, and current use of corticosteroids), (I) infection characteristics (i.e., time of acquisition, site of infection, and causative pathogen), (R) response characteristics (i.e., C-reactive protein, white blood cell count, temperature, respiratory rate, and heart rate), and (O) level of organ dysfunction at the time of prediction. We did not include composite markers of disease severity, such as the Simplified Acute Physiology Score (SAPS) or Acute Physiology and Chronic Health Evaluation (APACHE) score, since these have been formally defined only for a (first) 24-h observation window in the ICU, and were, therefore, considered less suitable for “real-time” bedside prognostication.

### Missing data

Patient characteristics (measured at baseline) were virtually complete, whereas 17% of daily physiological and laboratory values were missing overall (median 1%, range 0–80%, for individual variables), with > 50% missingness on daily measurement of activated partial thromboplastin time, albumin, alanine transaminase, aspartate transaminase, and lactate. Because longitudinal information was typically available, we performed trend imputations for a maximum duration of 2 days, according to methods as described by us previously [5]. As a consequence, the percentage of missing data was reduced to 11%. Of note, there were no missing data regarding discharge and death. We then used multiple imputation based on the information contained in all variables described in Table 3.

**Table 3:** Predisposition, infection, response, and organ failure (PIRO) characteristics of admissions stratified by admission status

Variable	At risk, *N* = 53	Limited organ failure, *N* = 1151	Multiple-organ failure, *N* = 167	*P* value
Predisposition				
Age (years)	63 (48–73)	63 (53–71)	63 (52–71)	0.90
Male gender	61 (61)	709 (61)	82 (62)	0.98
Chronic comorbidities				
Diabetes mellitus	26 (26)	217 (19)	24 (18)	0.19
Cardiovascular disease^a^	28 (28)	318 (27)	30 (23)	0.49
Immunodeficiency^b^	33 (33)	316 (27)	41 (31)	0.33
Renal insufficiency^c^	23 (23)	192 (16)	20 (15)	0.21
Respiratory insufficiency^d^	15 (15)	171 (15)	24 (18)	0.59
Admission type, medical	78 (78)	853 (73)	103 (77)	0.22
Insult				
Source (hospital-acquired)	42 (42)	514 (44)	65 (49)	0.51
Site/organ system				0.036
Pulmonary	68 (68)	676 (58)	61 (46)	
Abdomen	5 (5)	125 (11)	20 (15)	
Urinary tract	6 (6)	67 (6)	11 (8)	
Other or unknown	21 (21)	298 (26)	41 (31)	
Response				
SIRS criteria^e^				
Temperature	53 (53)	683 (59)	92 (69)	0.027
Leukocytes	65 (65)	839 (72)	93 (70)	0.32
Respiratory rate	86 (86)	1122 (96)	130 (98)	< 0.001
Heart rate	75 (75)	939 (81)	122 (92)	0.002
C-reactive protein (mg/L)	118 (75–209)	189 (101–296)	225 (123–293)	0.015
Lactate (mmol/L)	2.0 (1.3–2.9)	2.7 (1.7–4.5)	6.2 (4.1–10.7)	< 0.001
Organ dysfunction				
SOFA score at admission	5 (4–7)	8 (7–10)	12 (11–15)	< 0.001
APACHE IV score	70 (60–87)	84 (69–102)	112 (95–130)	< 0.001
Outcome				
ICU case fatality	5 (9)	180 (16)	67 (40)	< 0.001
ICU length of stay (days)	5 (3–12)	7 (3–12)	10 (4–18)	< 0.001

Data are numbers (percentage) or median (inter-quartile range)

*Abbreviations*: *APACHE* Acute Physiology and Chronic Health Evaluation, *ICU* intensive care unit, *SIRS* systemic inflammatory response syndrome

^a^Cardiovascular disease was defined as cerebrovascular disease or chronic cardiovascular insufficiency (New York Heart Association class 4), chronic congestive heart failure (ejection fraction < 30%), or peripheral vascular disease (intermittent claudication, patients with percutaneous transluminal angioplasty or bypass for arterial insufficiency)

^b^Immunodeficiency was defined as having acquired immune deficiency syndrome, the use of corticosteroids in high doses (equivalent to prednisolone of > 75 mg/day for at least 1 week), current use of immunosuppressive drugs, current use of antineoplastic, drugs recent hematologic malignancy, or documented humoral or cellular deficiency

^c^Renal insufficiency was defined as chronic renal insufficiency (creatinine > 177 μmol/L) or chronic dialysis

^d^Respiratory insufficiency was defined as chronic obstructive pulmonary disease or chronic respiratory insufficiency with functional disabilities (chronic mechanical ventilation, oxygen use at home, or severe pulmonary hypertension)

^e^Systemic inflammatory response syndrome criteria were defined as temperature < 36.0 or > 38.0 °C during at least 2 and 1 h, respectively; white blood cell count < 4 or > 12 × 10^9^/L or > 10% immature (band) forms; heart rate > 90/min during at least 1 h; respiratory rate > 20/min during at least 1 h, pCO_2_ < 32 mmHg, or mechanical ventilation

### Statistical analysis

**Fig. 1:**
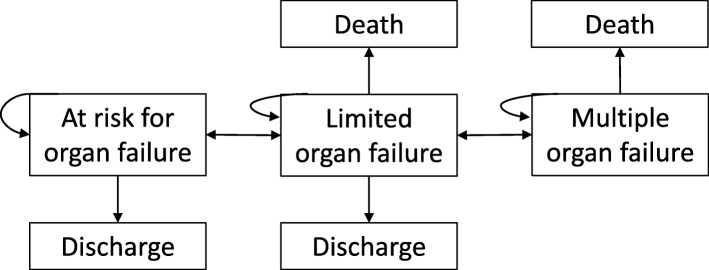
Proposed Markov model showing all possible transitions. The arrows represent forward or backward progression between transitional (disease severity) states, as well as to the final absorbing states death or discharge. The probabilities of advancing to a more advanced stage or regressing to a less severe stage or to an absorbing state are calculated by the multi-state Markov model with piecewise constant intensities. Forty-three out of a total of 3855 transitions (1%) were from “at risk” directly to “failure” or death or from “failure” directly to “at risk” or discharge and were not estimated due to the insufficient number of events

We estimated for each individual patient with sepsis the transition probabilities between the three transient states (at risk, severe organ dysfunction, and established multiple-organ failure) and the two absorbing states (discharge alive and death in ICU) (Fig. 1). Using these estimates, the absolute probabilities of the final absorbing states death, discharge, and established multiple-organ failure after 2 weeks of ICU admission were calculated.

To this end, we applied a continuous-time Markov multi-state model with piecewise constant intensities [6]. In essence, the model is similar to a multinomial logistic regression, but has the advantage of being able to produce transition probabilities for the prediction of disease progression with a more straightforward estimation of the standard error, to predict multiple outcomes, and to include new information on disease severity as it becomes available during ICU admission. A Markov model assumes that future transitions are dependent only on the current state variable. Carry-over effects may occur when values of predictor variables are affected by already “incubating” organ failure, and thus become part of the outcome rather than being a true prognostic factor. Transitions were, therefore, only modeled for every other day (days 1, 3, 5, etcetera until day 15). We focused on outcomes occurring during the first 2 weeks of admission only. By this, we prevented modeling outcomes that were no longer directly related to the sepsis episode present upon arrival in the ICU. Most deaths (78%) in our cohort occurred within the first 2 weeks, suggesting that indeed the majority of relevant outcomes was captured within this time window.

For model development, we first performed univariable analyses to examine associations between outcome and possible (a priori selected) predictors as described before. All predictors yielding a significant association (*P* value < 0.10) were then included in the final model. Due to highly computationally intensive analyses (typical runs took > 4 h), we did not perform any further selections such as backward or forward selection. Prognostic performance of the model was assessed using the c-statistic. Typically, in models predicting a dichotomous outcome, the c-statistic reflects how well a prediction rule can discriminate between patients who do or do not have the event (e.g., death). Good discriminatory ability is typically assumed at values > 0.7 [7]. However, when predicting multiple (mutually exclusive) outcome states, computation of a “simple” c-statistic is not feasible and therefore we used an alternative method, which summarizes the c-statistics of all separate transitions [8]. This c-statistic is a discrimination measure between states that was calculated using the predicted occupation probabilities. It counts the percentage of patients for whom the predicted occupation probability of being in, for instance, the state “at risk” is larger than the predicted probability of being in “persistent organ failure” at a particular time (averaged with the opposite transition), and it is also calculated for non-occurring transitions such as between discharge and death. Since the various transitions might be driven by different predictors, some transitions may have an unsatisfactory discrimination resulting in a lower (than expected) c-statistic. The Brier score was used to compare the prediction accuracy of a model including only baseline information to the same model which also included time-varying information [9]. The Brier score is a proper score function measuring the accuracy of probabilistic predictions. We applied the final model to the validation cohort and compared predicted probabilities to observed outcomes. The full prediction model is provided upon request.

Analyses were performed using R studio version 3.0.2 (R Core Team 2013, Vienna, Austria) [10] and SAS 9.2 (Cary, NC). The R-package msm [6] was used for implementation of the models. The SAS module “proc mi” was used for imputation (5 imputations using a random seed number and using all predictors). *P* values < 0.05 were considered to be statistically significant.

## Results

### Study population

**Fig. 2:**
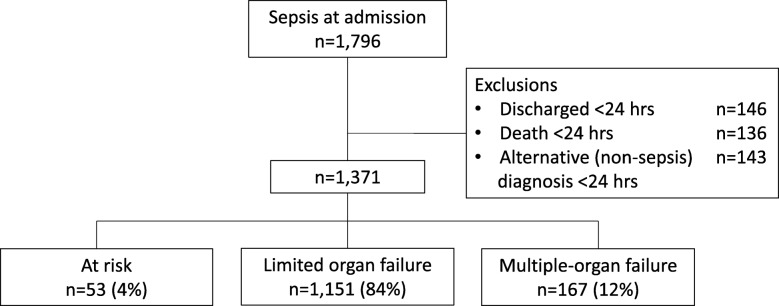
Flowchart of patient inclusion with patient disposition at admission

For model development, we studied 1371 ICU admissions for sepsis in 1251 patients, yielding 10,891 observation days. Eleven (0.80%) patients on palliative care were discharged alive from the ICU; 22 days of observation (0.2%) were therefore excluded from the analysis. ICU mortality by day 14 was 252 (18%), and total ICU mortality was 320 (23%). Figure 2 shows the classification of patients across the three categories of organ failure at the time of ICU admission. Among the 1151 admissions presenting with limited organ failure, 197 (17%) evolved to a more severe disease stage or died, 145 (13%) remained in the same stage, and 809 (70%) improved or were discharged alive by day 14. Among the 167 patients admitted with overt multiple-organ failure, 67 (40%) died, 91 (54%) improved or were discharged alive, and 6% remained in the ICU with organ failure beyond day 14. For comparison, 38 (72%) of the 53 patients who were considered to be at risk for organ failure were discharged within 14 days, and only 5 (9%) patients in this subgroup eventually died. Of note, all latter patients went through more severe stages of organ failure first. These descriptive results therefore indicate that our classification of organ dysfunction reflects both improvement and progression of disease well.

Age, gender, presence of chronic comorbidities, and admission type did not significantly differ between patients if stratified by the severity of organ failure present at admission (Table 3). However, length of stay was prolonged and case fatality higher in patients in whom multiple-organ failure was already overt upon ICU admission (Additional file 1: Figure S1). The evolution of organ dysfunction for the entire study cohort during the first 2 weeks in ICU is shown in Additional file 2: Figure S2. For all individual organ systems, dysfunction was most prevalent on day 1. Especially cardiovascular dysfunction improved over the first days in ICU, but other organ systems remained more or less stable during the first 2 weeks of admission.

### Univariable predictors of clinical trajectory

Additional file 3: Table S1 shows the crude hazard ratios for the various state transitions for potential defined predictor variables. Age, body mass index, immunocompromised state, renal insufficiency, respiratory insufficiency, site of infection, C-reactive protein, white blood cell count, fever, new onset atrial fibrillation, ICU-acquired onset of infection, bacteremia, and corticosteroid use were all included based on associations with any outcome in univariable analysis. The predictors gender, congestive heart failure, cardiovascular compromise, and causative pathogen were removed from the model since they were not significantly associated with any of the outcomes.

### Outcomes

**Fig. 3:**
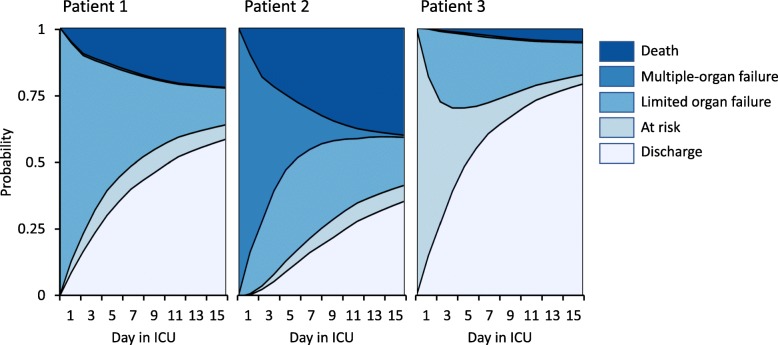
Modeled incidences of organ failure, death, and discharge in three illustrative patients. Patient 1 is a 72-year-old immunocompromised male admitted for a community-acquired pneumonia with mild hypoxemia (60% oxygen mask), a lactate level of 0.5 mg/L and a C-reactive protein level of 153 mg/L upon presentation. He has an absolute risk for discharge alive of 58% and death of 22% at day 14. Patient 2 represents another (but similar) patient with a community-acquired pneumonia in acute respiratory distress (requiring prompt intubation), hypotension (requiring norepinephrine), mottled skin, oliguria, lactate 4.2 mg/L, and a C-reactive protein of 268 mg/L. He has a risk for discharge alive of 36% and death of 40% at day 14. Patient 3 is a 53-year-old previously healthy female patient with a urinary tract infection, lactate of 0.4 mg/L, and a C-reactive protein of 50 mg/L. She has a probability of discharge alive of 79% and a probability of death of 5% at day 14

**Fig. 4:**
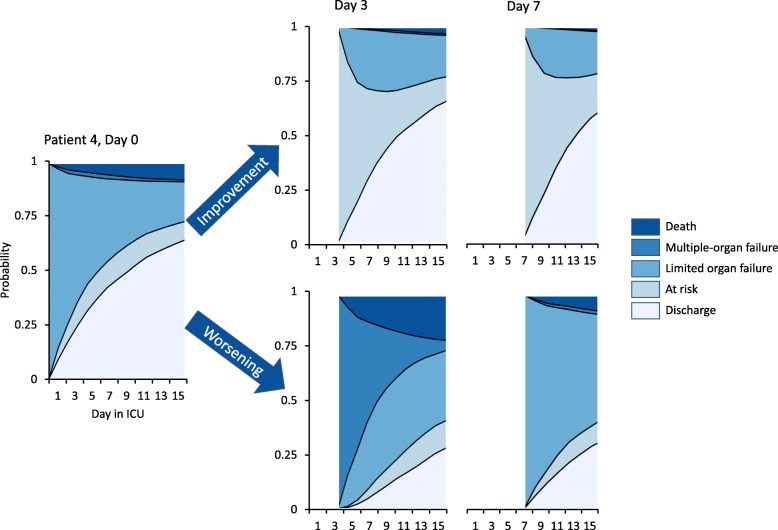
Outcome of patients who improve or worsen over time. Patient 4 is a 59-year-old male patient admitted for a severe peritonitis requiring noradrenaline at a rate of 0.05 μg/kg/min, a lactate level of 5.6 mmol/L, and a C-reactive protein level of 256 mg/L. At day 3, the noradrenaline can be stopped, his lactate levels are 0.5 mmol/L, and his C-reactive protein levels decrease to 170 mg/L (indicated by “improvement”), and at day 7, C-reactive protein levels dropped to 50 mg/L. However, if the same patient would develop refractory shock and atrial fibrillation at day 3, his outcome is as shown by “worsening”; at day 7, he develops an ICU-acquired pneumonia but noradrenalin is stopped, showing the net positive effect of worsening (pneumonia) and improvement (stopping of noradrenalin)

The c-statistic of our model in the derivation dataset was 0.67 (95% CI 0.63–0.70) overall, with c-statistics for individual daily state transitions ranging between 0.55 and 0.81. For example, the model predicted progression to established multiple-organ failure on day 14 quite well (c-statistic 0.77), whereas prediction of death proved more difficult (c-statistic 0.60). For comparison, the APACHE IV score was associated with mortality with a c-statistic of 0.68 (0.65–0.71). The Brier score was 0.64 for a baseline model and 0.60 for the model with time-varying information, yielding a 7.7% reduction of the prediction error. As an example of how the model can be used, Fig. 3 shows the evolution of organ failure and final outcomes for three individual patients as predicted on day 1 in the ICU. In addition, Fig. 4 (showing yet another subject) illustrates how the model may be used to generate updated predictions as the clinical condition of a patient improves or worsens over time.

### Model validation

Five hundred fifty-three patients were included in the validation cohort. Patient characteristics and the presence of organ failure upon ICU admission were similar as in the derivation cohort (Additional file 4: Table S2); 14 (2.5%) patients were classified at risk, 484 (88%) had organ dysfunction, and 55 (10%) established multiple-organ failure. ICU mortality was 91 (16%) by day 14 and 129 (23%) overall. The c-statistic of the model in this validation cohort was 0.66 (95% CI 0.62–0.70).

## Discussion

We developed a model to predict temporal changes in disease severity in critically ill patients presenting with sepsis to our ICU. The model estimates daily probabilities of progression or resolution of organ failure for individual patients, is updatable with new clinical information as it becomes available in the ICU, and can be used to predict the absolute risks of death, discharge, or remaining in the ICU. Although overall discrimination for our multi-state model was moderate based on a c-statistic of 0.66 (95% CI 0.62–0.70) in the validation dataset, it must be noted that this measure should not be directly compared to the reported AUCs of traditional regression models with a dichotomous outcome. Our model predicts five separate outcomes, and the c-statistic thus merely reflects an “average” accuracy for all of these. For example, discriminative ability for predicting transition to persisting organ failure was good, yet we observed less favorable accuracy for predicting death. In addition, predictive accuracy for mortality was similar to the widely used APACHE IV score.

With our approach, we aimed to develop a new modeling framework that uses daily updatable information, since outcome prediction is relevant not only on the first day of admission, but also later during ICU stay (i.e., once initial organ support has been provided). Disease severity may have changed considerably by then, and admission data might no longer be sufficiently current nor comprehensive to accurately predict outcome. In addition, the model not only predicts death, but also other important clinical outcomes such as occurrence of multiple-organ failure. Our model may thus assist clinicians during initial resuscitation as well as in later decision-making or to estimate the added prognostic value of novel biomarkers. We are aware of only as single other study that uses time-varying covariables to estimate the risk of sepsis progression during the first week in patients treated for infection [11]. They concluded that intraabdominal and respiratory sources of infection, independently of SOFA and APACHE scores, increased the risk of progression to more severe stages of sepsis. Of note, this study also enrolled less severely ill patients in hospital wards for whom predictions of clinical response might be very different.

Current sepsis-3 criteria categorize patients based on the dichotomized presence or absence of organ dysfunction. As a consequence, they do not provide detailed information about the severity of individual organ failures, nor their duration (and thus potential reversibility). To be able to model evolution of disease severity more accurately over time, we used a conceptual approach by which subjects were classified as being merely at risk of organ dysfunction, having established organ dysfunction, or having persisting multiple-organ failure. Although there is currently no commonly accepted way to accomplish this, we based our classification scheme on (an extended version of) the widely used SOFA score, but also considered the duration of individual organ failures.

We acknowledge some limitations of our study. First, this study was performed in two tertiary centers in the Netherlands and may thus not reflect general ICU practice in other settings. Both ICUs used selective digestive tract decontamination (SDD) throughout the study period, which may also limit generalizability of the study. Second, predictors were selected using univariable analysis, but further optimization of the model was not possible due to computer power constraints. Third, this model only predicts outcomes up to day 14 and might not be directly comparable to other studies with longer term outcomes. However, we opted for a shorter follow-up time to better capture the direct effects of sepsis occurring at admission; in addition, most discharges and deaths occurred before day 14 (78%). Fourth, we did not formally validate our definitions of organ dysfunction. However, we believe that this does distract neither from the face validity of the criteria used nor from the main study findings, since the purpose of this project was mostly to provide a new conceptual framework for modeling of clinical sepsis responses rather than a directly applicable prediction algorithm for clinical use. Finally, although we tested our model using prospectively collected independent data obtained in one of the two original study centers, it would have been better to validate our model externally.

## Conclusions

We propose a model that predicts daily evolution of disease severity in critically ill patients with sepsis and can be used to identify patients who will likely benefit most from aggressive interventions during the first 2 weeks in ICU. This model can also potentially be used to simulate the effects of new treatments, help in the design of new sepsis trials, and estimate the added prognostic value of novel biomarkers.

## Supplementary information

**Additional file 1:** **Figure S1.** Outcome of patients with sepsis stratified by severity of organ failure at admission. Representation of the length of stay and mortality of patients with sepsis admitted with low, intermediate and high levels of organ failure.

**Additional file 2:** **Figure S2.** Evolution of organ failure over time. Representation of the distribution of the severity of organ failure by organ system during the first 9 days of admission. Since central nervous system (CNS), renal and abdominal organ failure had to be present for > 1 day, patients could not be admitted with this type of organ failure. The last panel shows the level of organ failure and the absorbing states death and discharge on the patient level. For this panel, “at risk” was defined as moderate dysfunctions of limited duration in ≤2 organ systems; “limited organ failure” as moderate dysfunctions of limited duration in ≤3 organ systems, or severe dysfunctions in ≤2 organ systems, and “multiple-organ failure” as severe dysfunctions in ≥3 organ systems.

**Additional file 3:** **Table S1.** Transition hazard rates for selected variables. Representation of the crude hazard ratios for the various state transitions for several potential defined predictor variables in univariable analysis.

**Additional file 4: Table S2.** Predisposition, infection, response, and organ failure (PIRO) characteristics of patients stratified by level of organ failure at admission in the validation cohort. Data are numbers (percentage) or median (inter-quartile range). Abbreviations: APACHE Acute Physiology and Chronic Health Evaluation; ICU Intensive Care Unit; SIRS Systemic Inflammatory Response Syndrome.

## Data Availability

The datasets used and/or analyzed during the current study are available from the corresponding author on reasonable request.
