# Correction to: Predicting the clinical trajectory in critically ill patients with sepsis: a cohort study

**DOI:** 10.1186/s13054-020-2758-1

**Published:** 2020-02-06

**Authors:** Peter M. C. Klein Klouwenberg, Cristian Spitoni, Tom van der Poll, Marc J. Bonten, Olaf L. Cremer, on behalf of the MARS consortium, Marcus J. Schultz, Janneke Horn, Brendon Scicluna, Maryse A. Wiewel, Jos F. Frencken, Kirsten van de Groep, Marlies E. Koster-Brouwer, David S.Y. Ong, Diana Verboom, Friso M. de Beer, Lieuwe D. J. Bos, Gerie J. Glas, Roosmarijn T. M. van Hooijdonk, Laura R. A. Schouten, Marleen Straat, Esther Witteveen, Luuk Wieske, Arie J. Hoogendijk, Mischa A. Huson, Lonneke A. van Vught

**Affiliations:** 1 Department of Medical Microbiology and Immunology, Rijnstate Hospital, Wagnerlaan 55, 6815 AD Arnhem, the Netherlands; 2 Department of Mathematics, University Utrecht, Utrecht, the Netherlands; 3 Center for Experimental and Molecular Medicine, Division of Infectious Diseases, Amsterdam University Medical Centers, location Academic Medical Center, University of Amsterdam, Amsterdam, the Netherlands; 4 Department of Medical Microbiology, University Medical Center Utrecht, Utrecht University, Utrecht, the Netherlands; 5 Julius Center for Health Sciences and Primary Care, University Medical Center Utrecht, Utrecht University, Utrecht, the Netherlands; 6 Department of Intensive Care Medicine, University Medical Center Utrecht, Utrecht University, Utrecht, the Netherlands

**Correction to: Crit Care (2019) 23:408**

**https://doi.org/10.1186/s13054-019-2687-z**

In the publication of this article [1], there are 4 collaborating authors missing from the ‘MARS consortium’. This has now been included in this correction article.

Marcus J. Schultz Department of Intensive Care, Academic Medical Center Amsterdam, the Netherlands

Janneke Horn Department of Intensive Care, Academic Medical Center Amsterdam, the Netherlands

Brendon Scicluna Center for Experimental and Molecular Medicine, Academic Medical Center Amsterdam, the Netherlands

Maryse A. Wiewel Center for Experimental and Molecular Medicine, Academic Medical Center Amsterdam, the Netherlands
