# Supplementary figures and images for: Predicting the clinical trajectory in critically ill patients with sepsis: a cohort study
Source: Crit Care. 2019 Dec 12;23:408. doi: 10.1186/s13054-019-2687-z (PMC6909511; doi:10.1186/s13054-019-2687-z)

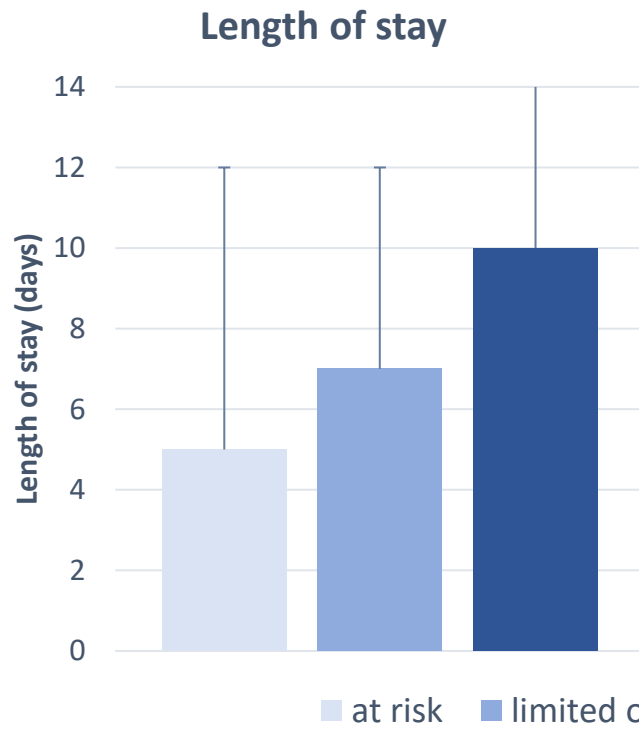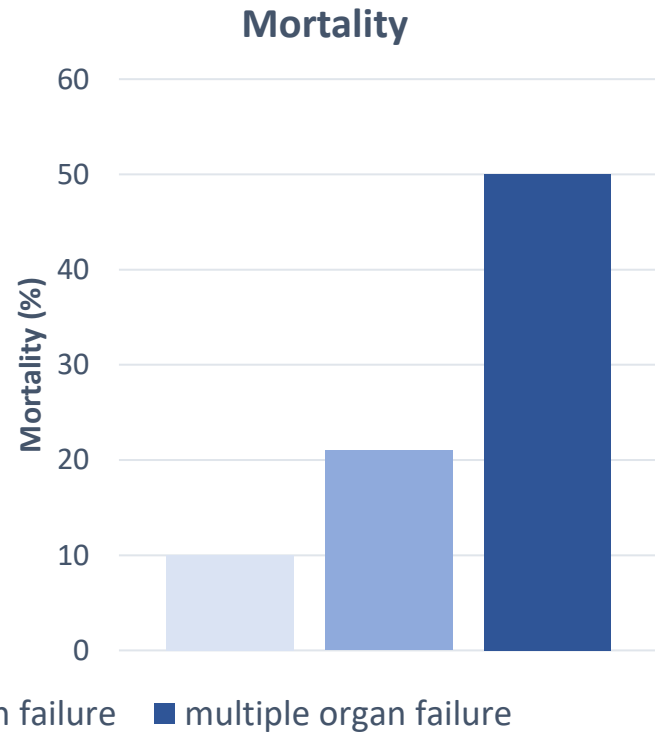

Supplement: Supplementary file 1 — Additional file 1: Figure S1. Outcome of patients with sepsis stratified by severity of organ failure at admission. Representation of the length of stay and mortality of patients with sepsis admitted with low, intermediate and high levels of organ failure. [file 13054_2019_2687_MOESM1_ESM.pdf]

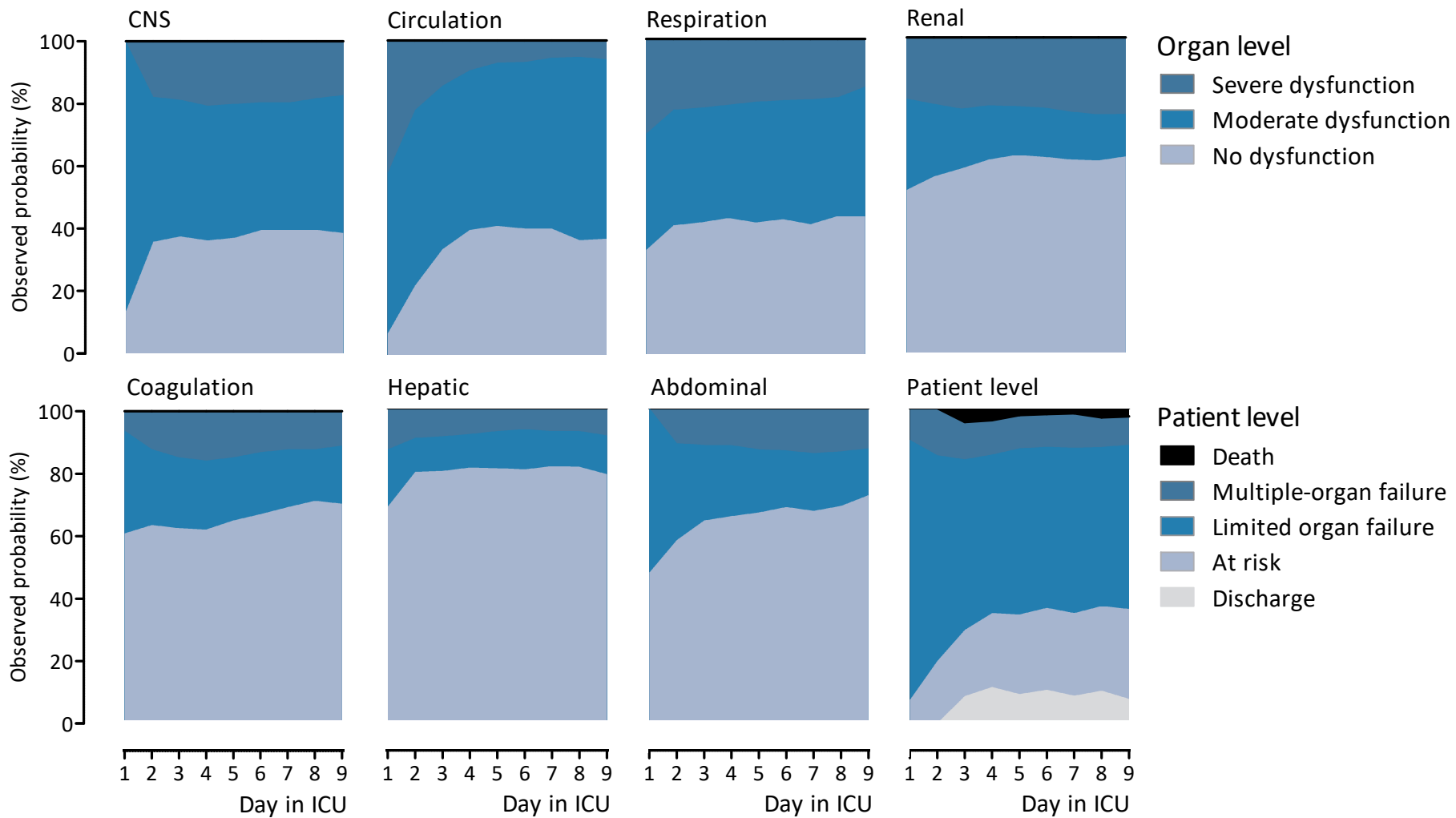

Supplement: Supplementary file 2 — Additional file 2: Figure S2. Evolution of organ failure over time. Representation of the distribution of the severity of organ failure by organ system during the first 9 days of admission. Since central nervous system (CNS), renal and abdominal organ failure had to be present for > 1 day, patients could not be admitted with this type of organ failure. The last panel shows the level of organ failure and the absorbing states death and discharge on the patient level. For this panel, “at risk” was defined as moderate dysfunctions of limited duration in ≤2 organ systems; “limited organ failure” as moderate dysfunctions of limited duration in ≤3 organ systems, or severe dysfunctions in ≤2 organ systems, and “multiple-organ failure” as severe dysfunctions in ≥3 organ systems. [file 13054_2019_2687_MOESM2_ESM.pdf]
