# Supplementary material for: Predicting the clinical trajectory in critically ill patients with sepsis: a cohort study
Source: Crit Care. 2019 Dec 12;23:408. doi: 10.1186/s13054-019-2687-z (PMC6909511; doi:10.1186/s13054-019-2687-z)
Supplement: Supplementary file 3 — Additional file 3: Table S1. Transition hazard rates for selected variables. Representation of the crude hazard ratios for the various state transitions for several potential defined predictor variables in univariable analysis. [file 13054_2019_2687_MOESM3_ESM.pdf]

Table S1: Transition hazard rates for selected variables

	At risk to		Limited organ failure to				Multiple-organ failure to	
**Variable**	**Discharge**	**Limited organ failure**	**Discharge**	**At risk**	**Multiple-organ failure**	**Death**	**Limited organ failure**	**Death**
Age (<60 = ref)								
60-80	0.93 (0.69-1.24)	1.23 (0.9-1.69)	1.03 (0.87-1.22)	1.06 (0.86-1.31)	1.00 (0.75-1.32)	1.32 (0.91-1.92)	1.12 (0.85-1.49)	1.46 (0.92-2.32)
>80	1.06 (0.66-1.70)	1.05 (0.6-1.83)	0.86 (0.61-1.21)	1.26 (0.89-1.77)	0.30 (0.11-0.82)	2.45 (1.46-4.14)	1.31 (0.64-2.69)	3.59 (1.30-9.95)
Immunodeficiency	0.87 (0.61-1.24)	1.19 (0.83-1.71)	1.06 (0.87-1.28)	0.78 (0.62-1.00)	1.18 (0.87-1.59)	1.06 (0.72-1.55)	0.84 (0.63-1.13)	1.17 (0.75-1.84)
Site of infection (abdomen = ref)								
Pulmonary	0.84 (0.59-1.19)	1.24 (0.85-1.80)	0.86 (0.68-1.09)	1.27 (0.99-1.62)	0.70 (0.45-1.09)	0.66 (0.41-1.05)	0.91 (0.6-1.40)	0.95 (0.50-1.79)
Urinary tract	1.01 (0.56-1.84)	1.19 (0.62-2.27)	1.25 (0.95-1.64)	0.63 (0.42-0.94)	1.4 (0.90-2.16)	0.57 (0.30-1.07)	0.69 (0.35-1.33)	0.97 (0.65-1.44)
C-reactive protein*	0.80 (0.56-1.14)	1.04 (0.73-1.48)	0.78 (0.66-0.94)	0.85 (0.69-1.06)	1.31 (0.99-1.74)	0.87 (0.61-1.24)	0.84 (0.65-1.08)	0.91 (0.61-1.38)
Bacteremia	1.32 (0.87-2.01)	0.75 (0.43-1.30)	1.02 (0.82-1.27)	0.70 (0.51-0.96)	1.49 (1.09-2.04)	1.11 (0.72-1.72)	0.80 (0.61-1.06)	0.95 (0.62-1.47)

*C-reactive protein levels were dichotomized using a cutoff of 100 mg/L. When levels decreased with more than 50 mmol/l compared to the previous measurement, the variable was set to 0. Only true bacteremias in post hoc analysis were included (contaminants were disregarded)
