# Supplementary material for: Predicting the clinical trajectory in critically ill patients with sepsis: a cohort study
Source: Crit Care. 2019 Dec 12;23:408. doi: 10.1186/s13054-019-2687-z (PMC6909511; doi:10.1186/s13054-019-2687-z)
Supplement: Supplementary file 4 — Additional file 4: Table S2. Predisposition, infection, response, and organ failure (PIRO) characteristics of patients stratified by level of organ failure at admission in the validation cohort. Data are numbers (percentage) or median (inter-quartile range). Abbreviations: APACHE Acute Physiology and Chronic Health Evaluation; ICU Intensive Care Unit; SIRS Systemic Inflammatory Response Syndrome. [file 13054_2019_2687_MOESM4_ESM.pdf]

Table S2: Predisposition, infection, response, and organ failure (PIRO) characteristics of patients stratified by level of organ failure at admission in the validation cohort

Variable	At risk<br><br>N=14	Limited organ failure<br>N=484	Multiple-organ failure<br>N=55	P-value
**Predisposition**				
Age	60 (50-70)	64 (54-73)	62 (54-69)	0.23
Male gender	8 (57)	322 (67)	39 (71)	0.60
Chronic comorbidities				
Diabetes mellitus	1 (7)	83 (17)	11 (20)	0.52
Cardiovascular disease [a]	3 (21)	152 (31)	18 (33)	0.71
Immunodeficiency [a]	5 (36)	159 (33)	18 (33)	0.97
Renal insufficiency [a]	3 (21)	65 (13)	10 (18)	0.46
Respiratory insufficiency [a]	2 (14)	114 (24)	6 (11)	0.08
Admission type, medical	10 (71)	361 (75)	40 (73)	0.17
**Insult**				
Source (hospital-acquired)	6 (43)	218 (45)	26 (47)	0.34
Site / organ system				0.06
Pulmonary	6 (43)	263 (54)	18 (33)	
Abdomen	2 (14)	46 (10)	13 (24)	
Urinary tract	0 (0)	5 (1)	0 (0)	
Other or unknown	6 (43)	170 (35)	24 (44)	
**Response**				
SIRS criteria [a]				
Temperature	6 (43)	273 (56)	33 (60)	0.51
Leukocytes	12 (86)	362 (75)	44 (80)	0.47
Respiratory rate	12 (86)	475 (99)	55 (100)	0.002
Heart rate	10 (71)	384 (79)	52 (95)	0.017
C-reactive protein	222 (60-354)	205 (113-306)	244 (147-371)	0.12
Lactate	2.3 (1.4-3.7)	2.4 (1.6-3.8)	6.5 (3.5-8.0)	<0.001
**Organ dysfunction**				
SOFA score at admission	5 (3-6)	8 (6-10)	12 (10-13)	<0.001
APACHE IV score	79 (56-89)	87 (71-108)	108 (91-127)	<0.001
**Outcome**				
ICU case fatality	0 (0)	107 (22)	22 (40)	0.001
ICU length of stay, day	3 (2-5)	8 (4-15)	15 (7-24)	<0.001

Data are numbers (percentage) or median (inter-quartile range). Abbreviations: APACHE Acute Physiology and Chronic Health Evaluation; ICU Intensive Care Unit; SIRS Systemic Inflammatory Response Syndrome. [a] Definitions of comorbidities and the systemic inflammatory response syndrome criteria are mentioned in table 3 of the article.
